# Unraveling the dual roles of tumor-infiltrating antibodies in solid tumors: friend or foe in the tumor microenvironment?

**DOI:** 10.3389/fimmu.2025.1671839

**Published:** 2025-11-21

**Authors:** Lingrui Miao, Run Zhou, Longfei Zhang, Linlu Feng, Yiao Wang, Pan Song, Dong Lv, Tai-Min Shen

**Affiliations:** 1 Department of Health Management & Institute of Health Management, Sichuan Provincial People’s Hospital, School of Medicine, University of Electronic Science and Technology of China, Chengdu, Sichuan, China; 2 Guizhou Medical University, Guiyang, Guizhou, China; 3 College of Pharmacy, Zhengzhou University, Zhengzhou, China; 4 Department of Urology, Institute of Urology, West China Hospital of Sichuan University, Chengdu, Sichuan, China; 5 Department of Urology, Deyang People’s Hospital, Deyang, Sichuan, China

**Keywords:** tumor-infiltrating antibodies, tumor microenvironment (TME), B cells, plasma cells, solid tumors

## Abstract

Within the tumor microenvironment (TME) of solid malignancies, tumor-infiltrating antibodies, have been identified as significant modulators of tumor progression and immune response. Tumor-infiltrating antibodies predominantly secreted by plasma cells but also including a small proportion of cancer-derived antibodies. This review aims to elucidate the multifaceted roles of tumor-infiltrating antibodies in the immunology of solid tumors, focusing on their dualistic nature within the TME. This review outlines the mechanisms of B cell activation, antibody class switching, plasma cell differentiation and antibody production, with a focus on their contributions to tumor immunity in solid cancers. Additionally, we discuss the emerging potential of tumor-infiltrating antibodies as both therapeutic targets and diagnostic biomarkers, offering insights that may inform future strategies in cancer treatment. Collectively, antibody functions are shaped by their isotypes: IgG is often associated with improved prognosis in various solid tumors. IgG1 and IgG3 generally mediate anti-tumor responses via antibody-dependent cell-mediated cytotoxicity (ADCC) and antibody-dependent cellular phagocytosis (ADCP), while IgG4 may impair immune effector functions and associate with immune tolerance. IgM, as an early humoral responder, enhances tumor surveillance through complement dependent cytoxicity (CDC), phagocytosis, and apoptosis induction. IgA predominantly promotes tumor progression through immune suppression. IgE exhibits context-dependent pro- and anti-tumor activities, though current evidence is limited, whereas the function of IgD remains largely unknown. Additionally, tumor-derived IgG promotes tumor growth, metastasis, and immune evasion. These findings may open new avenues of research to develop targeted therapies that modulate tumor-infiltrating antibodies, potentially improving the efficacy and safety profiles of current immunotherapeutic approaches. Overall, this review focuses on tumor-infiltrating antibodies in solid tumors and does not encompass hematological malignancies, aiming to provide a more precise understanding of antibody-mediated regulation within the solid tumor microenvironment.

## Graphical Abstract

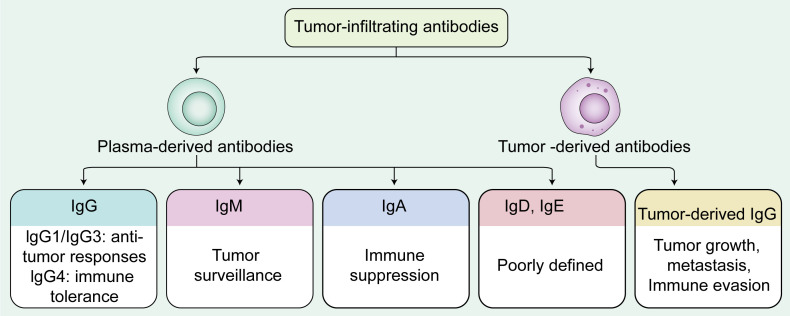

## 1 Introduction

Tumor microenvironment (TME) plays a crucial role in regulating tumor initiation, progression, and treatment responses (1). Historically, research into immune responses in cancer therapy has emphasized the centrality of cytotoxic T cells, particularly in the context of immune checkpoint inhibitors (ICIs). Despite achieving durable responses in some cancers, ICIs show limited efficacy in broader patient populations, with response rates as low as 10%–30% in various cancers (2, 3). These limitations, largely driven by resistance mechanisms, highlight the need for alternative or complementary strategies, such as leveraging humoral immunity in cancer therapy. Compared to T cell immunity, B cell-mediated humoral immunity in cancer remains underexplored. Humoral immunity is mainly participated by B cells, plasma cells, T follicular helper (Tfh) cells, etc., with the production and regulation of antibodies as the core response mechanisms. As immune interactions in the TME become clearer, B cells and their antibodies have emerged as key modulators of tumor progression and immunotherapy responses, shifting from passive participants to active players (4).

B cells within the TME exhibit diverse phenotypes and functional states, reflecting their complex and multifaceted roles in cancer immunity (5). Plasma cells—terminally differentiated B cells arising following antigen stimulation—serve as key effectors of humoral immunity. Their primary function is the secretion of antibodies, which aid in the recognition and clearance of antigens. In the context of cancer, tumor-infiltrating plasma cells display a dualistic nature. On one hand, they may enhance anti-tumor immune responses by producing tumor-specific antibodies and facilitating antigen presentation. On the other hand, they can contribute to immune suppression, chronic inflammation, and tumor immune evasion, depending on their isotype profile, spatial distribution, secretory activity, and the surrounding TME. These paradoxical roles underscore the need to dissect their context-dependent functions to better understand their prognostic and therapeutic potential.

Tumor-infiltrating antibodies are a crucial component of the immune response in cancer. Most of these antibodies are secreted by plasma cells, which are differentiated from B cells after encountering antigens. And a small percentage of antibodies are secretions from tumor cells. Despite increasing evidence supporting the involvement of B cells, plasma cells, and their antibodies in tumor immunity, their precise mechanisms of action remain unclear due to the complexity of the TME (6, 7). The roles of antibodies produced by tumor-infiltrating plasma cells are highly context-dependent, varying with both the characteristics of the TME and the specific isotype or subclass of the antibody. Depending on these factors, such antibodies may either promote or suppress anti-tumor immunity. Given the specificity and potency of these antibodies in modulating immune responses, targeting them in cancer therapy holds vast potential. Consequently, research on the role of antibodies in the TME has garnered significant attention, particularly with the expansion of antibody-based therapies. In addition to plasma cell-derived antibodies, emerging evidence suggests that tumor cells themselves can also produce Igs, commonly referred to as cancer-derived antibodies (8). These antibodies differ from those secreted by plasma cells and have been implicated in tumor progression, immune evasion, and metastasis (9). The coexistence of plasma cell-derived and tumor cell-derived antibodies within the TME introduces additional complexity to the humoral immune landscape in cancer. Different immunoglobulin (Ig) subclasses, including IgG, IgA, IgM, and IgE, exhibit distinct functions in various cancers, influencing immune responses, tumor cell interactions, and therapeutic efficacy. However, a systematic and comprehensive understanding of their roles is still lacking. Therefore, the aim of this review is to summarize current findings on tumor-infiltrating antibodies, discussing their roles in tumor immunity, their interactions within the TME, and their potential implications for immunotherapy.

## 2 Basic functions and classification of B cells

**Figure 1:**
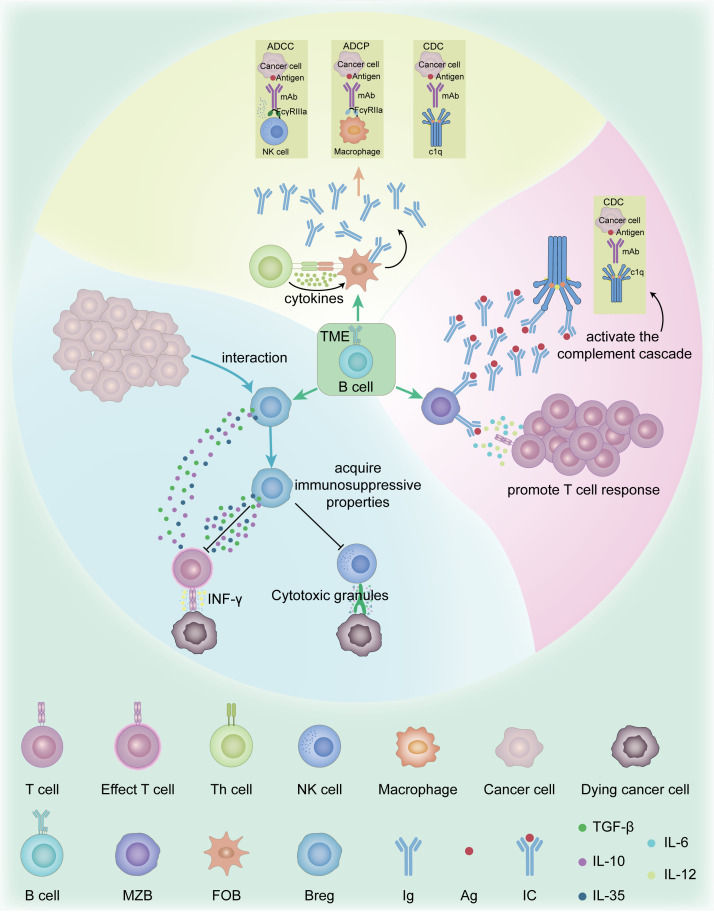
Function and mechanism of B cell subsets in the TME. The main types of B cells in the TME include FOB, MZB, and Bregs. FOB cells are primarily responsible for mediating T cell-dependent immune responses. In contrast, MZB cells are specialized for rapid T cell-independent responses. FOB cells contribute to anti-tumor immunity via antigen presentation and differentiation into antibody-producing plasma cells, which primarily secrete high-affinity antibodies. These antibodies engage immune effector mechanisms such as ADCC, ADCP, CDC. MZB cells, primarily secreting low-affinity antibodies, can exert anti-tumor effects by activating the complement cascade and promoting T cell responses. Bregs secrete anti-inflammatory cytokines such as IL-10, IL-35, and TGF-β, which suppress CTLs and NK cells functions, including IFN-γ secretion and granule-mediated cytotoxicity. ADCC, Antibody-dependent cell-mediated cytotoxicity; ADCP, Antibody-dependent cellular phagocytosis; Breg, Regulatory B cell; CDC, Complement dependent cytoxicity; CTLs, Cytotoxic T Lymphocytes; FOB, Follicular B cell; IFN-γ, Interferon Gamma; IL-10, Interleukin-10; IL-35, Interleukin-35; MZB, marginal zone B cells; NK cells, Natural killer cells; TGF-β, Transforming Growth Factor Beta; TME, Tumor microenvironment.

B cells play complex and diverse roles in the TME. According to existing literature, the main types of B cells in the TME include follicular B cells(FOB), marginal zone B cells (MZB), and regulatory B cells (Bregs) (10, 11). FOB cells are mainly involved in T cell-dependent immune responses, whereas MZB cells play an important role in T cell-independent immune responses (12). FOB cells contribute to anti-tumor immunity via antigen presentation and differentiation into antibody-producing plasma cells, which primarily secrete high-affinity antibodies. These antibodies engage immune effector mechanisms such as antibody-dependent cell-mediated cytotoxicity (ADCC) (13), antibody-dependent cellular phagocytosis (ADCP) (14), and complement dependent cytoxicity (CDC) (15). MZB cells, primarily secreting low-affinity antibodies, can exert anti-tumor effects by activating the complement cascade and promoting T cell responses (13). Bregs suppress anti-tumor immune responses and promote tumor growth and progression by secreting anti-inflammatory cytokines such as IL-10, TGF-β, and IL-35 (16). Bregs in the TME can acquire immunosuppressive properties through interactions with malignant cells, thereby inhibiting the functions of effector T cells and natural killer (NK) cells (16, 17). It can be seen that B cells play a dual role in the TME. On one hand, B cells can exert anti-tumor effects through antigen presentation and antibody production. On the other hand, regulatory B cells can promote tumor growth through their immunosuppressive functions (**Figure 1**).

## 3 B cell activation and antibody production

### 3.1 B cell activation and antibody class switching

B cells are activated upon antigen encounter through coordinated events, including B cell receptor (BCR) signaling, interactions with Tfh cells, transcription factor regulation, and metabolic reprogramming. Antigen binding to the BCR triggers signaling cascades that facilitate internalization and processing of the antigen, which is presented on MHC II to Tfh cells (18). This interaction, involving CD40-CD40L and IL-21 signaling, is crucial for B cell activation, proliferation, and differentiation (19). Metabolic reprogramming is also crucial during B cell activation. Upon activation, B cells shift from a quiescent to a highly proliferative state, requiring increased nutrient uptake and metabolic activity to support rapid cell division and antibody production (20, 21).

Antibody class switching, or class switch recombination (CSR), is a critical process initiated after B cell activation that enables the production of different antibody isotypes while preserving antigen specificity. Naive B cells initially express membrane-bound IgM and IgD, encoded by the constant region genes Cμ and Cδ respectively. Upon antigen encounter, activated B cells in germinal centers (GCs) upregulate activation-induced cytidine deaminase (AID), which induces double-strand breaks at the switch (S) regions upstream of the immunoglobulin heavy chain constant (C) region genes. These breaks are repaired via non-homologous end joining, resulting in the recombination of the V(D)J exon with downstream C genes (Cγ, Cα, or Cϵ) to generate IgG, IgA, or IgE isotypes, respectively (22, 23). Once CSR is complete, B cells acquire a fixed isotype profile and, upon terminal differentiation into plasma cells (PCs), inherit and secrete high levels of the corresponding antibody class. This process is tightly regulated by the cytokine milieu: IFN-γ promotes switching to IgG2, IL-4 to IgG4 and IgE, and TGF-β to IgA. Antibodies are functionally categorized into five major classes—IgM, IgD, IgG, IgA, and IgE—each defined by distinct constant region sequences that determine their effector functions in immune responses (24).

### 3.2 Generation of plasma cells and antibody production

Plasma cell generation begins in GCs of secondary lymphoid organs, where B cells undergo somatic hypermutation (SHM) and affinity maturation. High-affinity B cells in the GC light zone receive signals from antigens, initiating their differentiation into plasma cells. The completion of differentiation and migration of plasma cells from the dark zone to the GC exit depends on signals from Tfh cells (25). B cell differentiation involves several transcription factors, including Blimp-1, IRF4, and XBP1, which regulate gene expression and cell fate at various stages (26).

Plasma cell antibody secretion involves transcription, translation, folding, modification, and secretion. It depends on the endoplasmic reticulum and Golgi apparatus. The endoplasmic reticulum synthesizes and folds antibodies, while the Golgi apparatus modifies and transports them. In the Golgi, antibodies are packaged into vesicles and transported to the cell membrane via SNARE protein Sec22b for secretion (27). Efficient secretion also depends on plasma cells’ metabolic and protein homeostasis pathways, supported by metabolic activity and the unfolded protein response (UPR) (28).

### 3.3 The role of plasma cells in the tumor microenvironment

The distribution and abundance of plasma cells in the TME are highly heterogeneous, differing between tumor types and patients, and this variability results in diverse functions (4, 7, 29). Mature TLS often contain germinal center B cells and IgG-secreting plasma cells, and plasma cells frequently localize to or surround TLS in multiple tumor types. Indeed, studies in endometrial cancer, gastric cancer, bladder cancer, and pancreatic cancer have confirmed that high densities of plasma cells can be detected within or adjacent to TLS in the tumor microenvironment (30–37). TLS are ectopic lymphoid organs that form in non-lymphoid tissues at sites of chronic inflammation, such as tumors (38). TLS are observed in various tumor types, and are linked to anti-tumor immune responses and clinical outcomes (39, 40).

Within TLS, plasma cells primarily exhibit anti-tumor activity, whereas in the absence of TLS or upon direct interaction with tumor cells, they may promote tumor growth. Nonetheless, in some cases, TLSs may serve as a niche for hidden tumor cells and indicate a bad prognosis (41). Multiple studies have demonstrated that in tumors with mature TLS, plasma cells contribute to favorable clinical outcomes. For instance, in renal cell carcinoma, spatial transcriptomics revealed that TLS-containing tumors harbor IgG-producing plasma cells whose presence correlates with apoptotic malignant cells and improved progression-free survival (42). Similarly, in high-grade serous ovarian cancer, tertiary lymphoid structures are frequently surrounded by plasma cells expressing oligoclonal IgG, and these PCs are associated with robust CD8^+^ TIL responses and superior prognosis (43). In gastric cancer, TLS characterized by long-lived plasma cells also correlate with enhanced humoral responses and better outcome (44). These findings suggest that plasma cells in TLS often play antitumor roles. On the contrary, immature or “early” TLS that lack germinal centers fail to support full B cell activation and instead favor immunosuppressive circuits characterized by the upregulation of IL-10 and TGF-β (45). In settings where TLS are immature or absent, or upon direct tumor cell interaction, immunosuppressive or tumor-promoting functions of plasma cells may predominate, depending on cancer type and immune microenvironment. Additionally, plasma cells can form immune complexes through antibody secretion, activating macrophages and neutrophils, which contribute to chronic inflammation and a tumor-promoting environment (4, 40, 41).

## 4 Plasma cell-derived antibodies in tumor microenvironment

**Figure 2:**
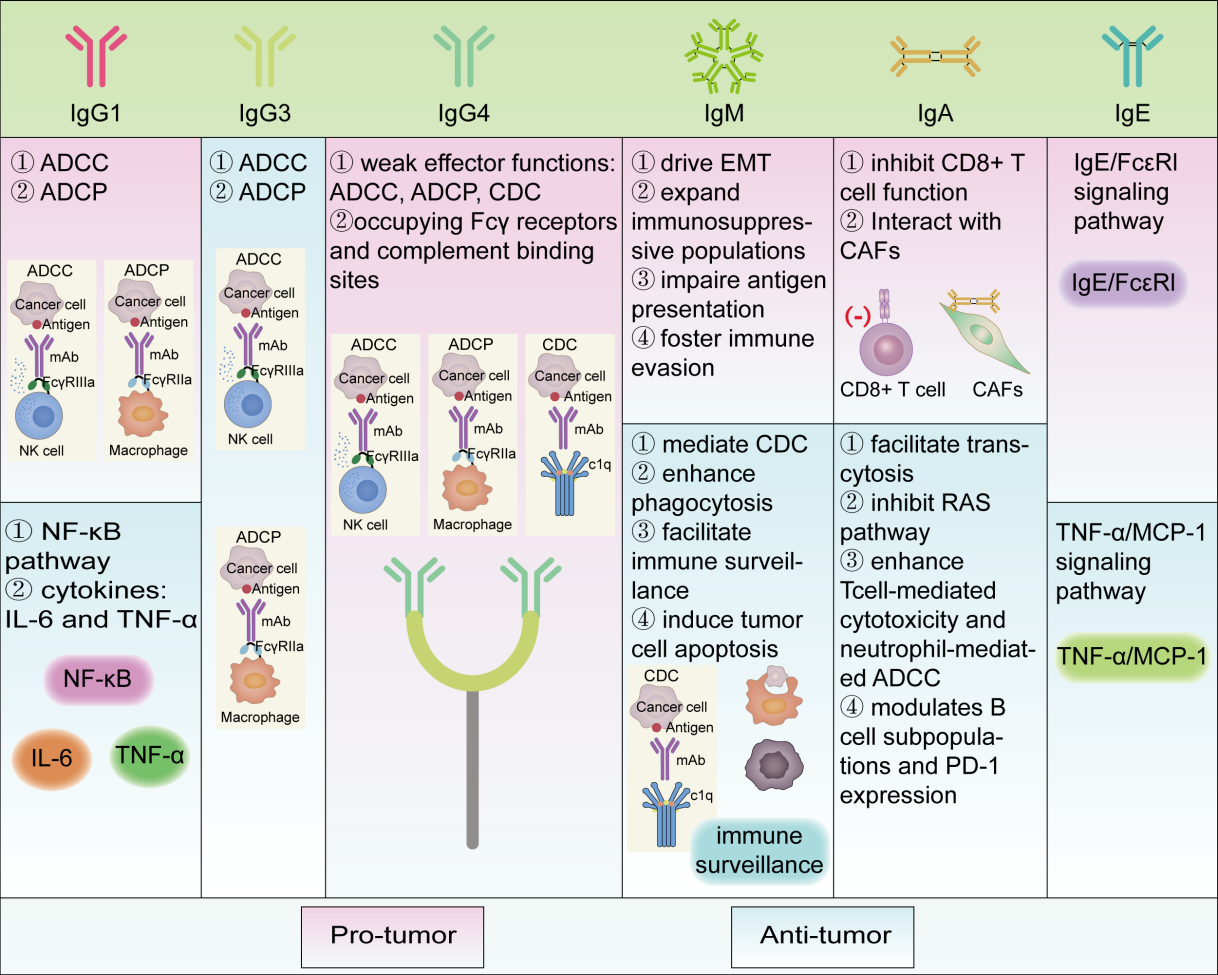
Different roles of various immunoglobulins in tumor immunity and their pro/anti-tumor mechanisms. Functional classes are indicated by different colored boxes in the figure, with pink representing pro-tumor and green representing anti-tumor. Most of the antibody subtypes play dual functional roles, whereas IgG3 and IgG4 are respectively anti-tumor and pro-tumor in the literature we reviewed. ADCC, Antibody-dependent cell-mediated cytotoxicity; ADCP, Antibody-dependent cellular phagocytosis; CD, Cluster of differentiation; CAFs, Cancer-associated fibroblasts; CDC, Complement dependent cytoxicity; EMT, Epithelial-mesenchymal transition; FcϵRI, Fc epsilon Receptor I; Ig, Immunoglobulin; IL-6, Interleukin-6; MCP-1, Monocyte Chemoattractant Protein-1; NF-κB, Nuclear transcription factor-κB; PD-1, Programmed Cell Death Protein 1; RAS, RAt Sarcoma; TNF-α, Tumor necrosis factor alpha.

The role of Ig secreted by plasma cells in tumors is a complex area, with recent studies revealing that these immunoglobulins can both promote and inhibit tumor growth in the TME (46). The following discussion is based on five different types of Igs (**Table 1**; **Figure 2**).

**Table 1:** Roles of different types antibodies secreted by plasma cells in different diseases.

Disease type	Antibody type	Specimen type	Species origin	Effect (P/I)	Introduction to the mechanism	Reference
Circulatory system						
Burkitt lymphoma	IgD	Cell line	Human	Promote	IgD activates IgDR to initiate the tyrosine phosphorylation signaling cascade to induce the expression of cyclin D3, CDK6, c-myc and inhibit P16INK4a, thereby accelerating the G1/S transition and promoting Daudi cell cycle progression.	(47)
Chronic lymphocytic leukemia	IgM	Serum	Mouse	Promote	sIgM promotes tumor progression by inducing the accumulation of myeloid-derived suppressor cells.	(48)
IgD multiple myeloma	IgD	Bone marrow, blood	Human	Promote		(49)
T-cell acute lymphoblastic leukemia (T-ALL)	IgD	Plasma, cell line	Human, mouse	Promote	IgD interacts with T cells through its Fc receptor, induces excessive proliferation of T-ALL cells and inhibits their apoptosis	(50)
	IgD	Cell line	Human	Inhibit	IgD interacts with T cells through Fc receptor, inducing excessive proliferation of T-ALL cells and inhibiting their apoptosis.	(50)
Digestive system						
Colon cancer	IgG4	Tumor tissue	Human	Promote	Induce tolerogenic M2-like macrophages via FcγRI and PI3K/AKT/STAT3 signaling, leading to an immunosuppressive tumor microenvironment	(51)
	IgM	Tumor tissue	Human	Inhibit	Innate immune surveillance; activates complement-dependent cytotoxicity (CDC); enhances innate immune response	(52)
	IgM	Tumor tissue	Mouse	Inhibit	Polyreactive IgM derived from L2pB1 cells is a key factor in cancer cell recognition, tumor growth inhibition, cancer cell death induction and clearance	(53)
Colorectal cancer with liver metastases	IgA	Tumor tissue	Human	Promote		(54)
Colorectal cancer	IgG	Cell line	Mouse	Promote	Promote tumor cell proliferation; reduce cancer cell apoptosis by inhibiting apoptosis-related pathways; enhance invasion and metastasis capabilities by promoting extracellular matrix degradation and cell migration	(55)
	IgG	Tumor tissue	Human	Promote	With a novel sialylated modification in Asn162 of CH1, was widely expressed in cancer stem cells of epithelial cancers, and promoted tumor progression via activating integrin-FAK signaling.	(56)
	IgG4	Tumor tissue	Human	Promote	Inhibits effector cell activity; competitively inhibits the anti-tumor effects of other IgGs; regulates the immunosuppressive microenvironment	(57)
	IgG	Tumor tissue	Human	Inhibit		(55)
	IgA	Tumor tissue	Human	Inhibit		
	IgA	Tumor tissue	Human	Promote		(58)
	IgA	Intratumoral immune cells	Human, Mouse	Inhibit	FMD abolished the inhibitory effect on CD8+ T cells by reducing the class-switch recombination of B cells to IgA, as FMD drove fatty acid oxidation metabolism in B cells.	(59)
	IgA	Serum	Human	Promote	It seems justified to conclude that elevation of circulating SIgA and SIgM in colorectal cancer patients strongly suggests liver metastasis.	(53)
	IgM			Promote		
	IgA	Serum	Human, Mouse	Inhibit	Targeting Erbin greatly suppressed lung metastasis of CRC by inhibiting PD1 expression of IgA+ B cells, promoting aggregation of IgA+ B cells, and increasing the killing effects of CD8+ T cells on tumor cells.	(60)
Esophageal cancer	IgG4	Tumor tissue	Human	Promote	Inhibits effector cell activity; competitively inhibits the anti-tumor effects of other IgGs; regulates the immunosuppressive microenvironment	(61)
Gastric cancer	IgA	Cell line	Mouse	Promote	IgA PCs were mainly involved in the regulation of phagocytic pathways, such as phagocytosis, and engulfment	(62)
Hepatocellular carcinoma	IgM	Serum, tumor tissue	Mouse	Promote	IgM promotes tumor metastasis through epithelial-mesenchymal transition mediated by polymeric immunoglobulin receptor	(63)
	IgM	Serum, tumor tissue	Human	Inhibit		(64)
	IgA	Blood, tumor tissue	Human, mouse	Promote	IgA+ cells induced by inflammation inhibit the activity of T cells by secreting specific immune regulatory factors, thereby weakening the body’s immune surveillance of liver cancer cells.	(65)
Pancreatic cancer	IgM	Tumor tissue	Human	Inhibit	Innate immune surveillance; activates complement-dependent cytotoxicity (CDC); enhances innate immune response	(52)
Gynecological and breast cancer						
Breast cancer	IgG	Serum	Mouse	Promote	Pathogenic IgG targeting glycosylated membrane HSPA4 selectively promotes lymph node metastasis and activates the downstream Src/NF-κB in ITGB5 and tumor cells for CXCR4/SDF1α axis-mediated metastasis.	(66)
	IgM	Serum	Human	Inhibit	Destroy circulating or seeded isolated disseminated tumor cells (micrometastases) that eventually could lead to metastatic disease and death.	(67).
	IgM	Tumor tissue	Human	Promote	As Breg cells prevent CSR by inhibiting the Tfh/IL21 axis, long-lived plasma cell generation is halted, resulting in lower IgG and a higher fraction of B cells unable to undergo CSR events, resulting in higher IgM.	(68)
Medullary ductal breast cancer	IgG	Tumor tissue	Human	Promote		(69)
Triple negative breast cancer	IgG	Tumor tissue	Human	Inhibit	Through ADCC and CDC, it also reduces tumor proliferation and metastasis potential by inhibiting tumor-promoting cytokines and signaling pathways.	(70)
	IgG4	Tumor tissue	Human	Promote	Immunosuppressive effects to promote tumor immune escape; inhibit the activation of cytotoxic T cells.	(71)
Endometrial cancer	IgA	Tumor tissue	Human	Promote	IgA activates inflammatory pathways within tumor cells by binding to polymeric immunoglobulin receptors (pIgR) on tumor cells.	(72)
Ovarian cancer	IgG4	Tumor tissue, cell line	Human, Mouse	Inhibit		(71)
	IgA	Tumor tissue	Human	Inhibit	Transcytosis of IgA sensitize tumor cells to cytotoxic killing by T cells.	(73)
	IgA	Cell line	Mouse	Inhibit	IgA-coated bacteria, a character in OC, was necessary for B-cell activation and for delaying the progression of TRAF3KO tumors.	(73)
	IgE	Tumor tissue, blood and tumor specimens	Rat, human	Inhibit	IgE antibodies recruit macrophages through the TNFα/MCP-1 signaling pathway, enhance anti-tumor immune responses.	(74)
Nervous system						
Neuroblastoma	IgM	Peripheral blood	Human	Inhibit	Direct cytotoxicity; activate immune effector cells; enhance immune response	(75)
	IgA	Cell line, tumor tissue	Human, mouse	Inhibit	IgA GD2 antibodies effectively kill tumor cells by activating neutrophil-mediated ADCC.	(76)
Respiratory system						
Lung cancer	IgG1	tumor tissue, adjacent tumor sites, blood	Human	Inhibit	Mainly through ADCC and CDC	(77)
	IgG3	tumor tissue, adjacent tumor sites, blood	Human	Inhibit	Mainly through ADCC and CDC	(77)
	IgM	Tumor tissue	Human	Inhibit	Innate immune surveillance; activates complement-dependent cytotoxicity (CDC); enhances innate immune response	(52)
Non-small cell lung cancer	IgG1	Tumor tissue	Human	Inhibit	Mainly through ADCC and CDC	(78)
	IgG3	Tumor tissue	Human	Inhibit	Mainly through ADCC and CDC	(78)
Urogenital system						
Bladder cancer luminal papillary (LumP) subtype	IgA	Tumor tissue	Human	Promote	Activate inflammatory response pathways and cytokine secretion.	(79)
	IgG1	Tumor tissue	Human	Promote	Activate inflammatory response pathways and cytokine secretion.	(79)
Bladder cancer luminal unstable (LumU) subtype	IgA	Tumor tissue	Human	Promote	Activate inflammatory response pathways and cytokine secretion.	(79)
	IgG1	Tumor tissue	Human	Inhibit	Enhance T-cell responses	(79)
Bladder cancer basal/squamous (Ba/Sq) subtype	IgA	Tumor tissue	Human	Inhibit		(79)
	IgG1	Tumor tissue	Human	Inhibit	Enhance T-cell responses	(79)
Bladder cancer	IgG1/IgA	Tumor tissue	Human	inhibit	A more prominent expression of costimulatory CD80, increased IL21-mediated signaling, checkpoint regulation, Fcg receptor signaling, and receptor-mediated phagocytosis and endocytosis.	(80)
Prostate cancer	IgG	Serum	Human	Promote		(81)
Skin						
Cutaneous tumors	IgE	Epithelial cells	Mouse	Promote	IgE/FcϵRI signaling promotes epithelial cell growth and differentiation in basophils, strongly driving tumor growth of epithelial cells carrying oncogenic mutations.	(74)
Melanoma	IgG	Serum	Human	Inhibit		(82)
	IgG4	Serum	Human	Promote	Inhibit antibody effector function; hinder effective antibody function; promote the formation of an immunosuppressive microenvironment	(83)
	IgM	Cell line, serum	Human	Inhibit	Eliminate subclinical tumor deposits in patients either before or after surgery for stage II melanoma.	(84)
	IgM	Serum	Human	Inhibit		(85)
	IgM	Myeloid cells	Mouse	Promote	Control BCR signaling, B2 cell development and function, and Th17 cell production of inflammatory cytokines.	(86)
	IgA1	Tumor tissue	Human	Promote	Propensity for CDRH3 in IgG1, IgG2 and IgA1 supports a highly active yet perturbed B cell compartment at the tumor site.	(87)
	IgG	Tumor tissue	Human	Promote		
Head and neck						
Squamous cell carcinoma of the head and neck	IgG	Serum	Human	Inhibit		(88)

### 4.1 IgG

IgG has been reported to play a dual role in the TME. Tumor-reactive IgG targets tumor-associated antigens and correlates with improved prognosis in several cancers. For example, in colorectal cancer, a high IgG+ plasma cell signature is linked to better overall survival (OS) (55). Similarly, in triple-negative breast cancer (TNBC), IgG is associated with enhanced survival, exhibiting clonal expansion and antigen-driven properties (70). Additionally, melanoma-specific IgGs, such as MelanA/MART-1 IgG1 and gp100 IgG2, are linked to prolonged OS (82). Enhanced IgG/IgM responses are observed in HPV-associated head and neck squamous cell carcinoma patients benefiting from PD-1 blockade (88).

Conversely, IgG can also promote tumor progression. Increased IgG levels in primary melanoma and skin metastases also implicate IgG is associated with tumor progression (87). In medullary ductal breast cancer, 70% of infiltrating plasma cells are IgG type, unlike IgA-dominant normal epithelium, with higher IgG levels promoting tumor progression (69). Pathogenic IgG targeting the glycosylated membrane protein HSPA4 has been shown to selectively promote lymph node metastasis in breast cancer patients by activating the HSPA 4 binding protein ITGB5 and the downstream Src/NF-κB pathway, which mediates metastasis via the CXCR 4/SDF-1 α axis. Additionally, elevated serum levels of anti-HSPA 4 IgG in breast cancer patients correlate with high tumor HSPA 4 expression and poor prognosis (66). Fetuin-A-specific IgG serves as a potential marker for metastatic prostate cancer (81), while also indicating its pro-tumorigenic role in disease progression.

#### 4.1.1 Subtypes of IgG and their role in tumors

IgG consists of four subclasses ranked by relative abundance in serum: IgG1, the most abundant, followed by IgG2, IgG3, and IgG4, participating in tumor immune regulation within local tissues (89).

#### 4.1.2 IgG1 and IgG3

IgG1 and IgG3 generally exhibit the highest affinity for Fcγ receptors (FcγR) and serve as potent mediators of ADCC and ADCP, both of which are crucial for antitumor immunity (90). Both contribute to antitumor immunity across various cancer types primarily via their efficient immune effector functions. However, their specific mechanisms of action and clinical significance vary depending on tumor type and microenvironment, providing critical insights for developing IgG1- and IgG3-based therapeutic strategies.

A single-cell RNA-seq and TCR-seq analysis from IIIA non-small cell lung cancer (NSCLC) patients revealed that neoadjuvant immunotherapy combined with chemotherapy enhanced IL-21 secretion from infiltrating Tfh cells, which subsequently promoted B cell class switching and plasma cell differentiation. As a result, plasma cell subsets expressing IgG1 and IgG3 were significantly enriched in the TME. These antibody isotypes may contribute to anti-tumor immunity in NSCLC by enhancing antibody-dependent cellular cytotoxicity ADCC and CDC (78). In lung cancer, levels of IgG1 and IgG3 antibodies are positively correlated with CD16a expression, suggesting that increased levels of these subclasses may enhance NK cell activation, as CD16a is a key activating receptor on NK cells (77). Single-cell RNA-seq analysis of bladder cancer patients revealed that IgG1 plasma cells predominated in the tumor immune microenvironment (TIME), exhibiting primarily oncogenic functions and strong correlations with inflammatory response pathways. These pathways, including the NF-κB signaling pathway, are associated with immune cell activation and the promotion of an inflammatory tumor microenvironment. Furthermore, IgG1 plasma cells were shown to promote the secretion of key inflammatory cytokines, including TNF-α, IL-6, and IL-10, which not only sustain immune responses but also contribute to immune evasion and tumor progression (79). Conversely, another bladder cancer study has identified high IgG1/IgA ratios predicts better survival outcomes, which are associated with elevated CD80 expression, enhanced IL-21-mediated signaling, checkpoint modulation, FcγR signaling, and receptor-mediated phagocytosis and endocytosis (80).

Collectively, IgG1 and IgG3 subclasses have been shown to exert anti-tumor effects in most cancer contexts, primarily by serving as effective mediators of ADCC and ADCP. However, in some contexts, IgG1+ plasma cells have also been associated with pro-tumorigenic effects, potentially through their involvement in inflammatory response pathways such as NF-κB signaling and secretion of cytokines like IL-6 and TNF-α, thereby contributing to immune evasion and tumor progression (**Figure 2**).

#### 4.1.3 IgG2

IgG2 binds weakly to FcγR, minimally activates NK cells and macrophages, exhibits reduced ADCC and CDC activity, and demonstrates a lower anti-tumor effect (91). There are few reports on the role of IgG2 in the TME.

#### 4.1.4 IgG4

IgG4 is the least abundant IgG subclass in human serum, with the lowest levels among IgG subclasses in most tumor patients (89). IgG4 has a short hinge region, limited Fab arm flexibility, and weak antigen binding. It interacts minimally with FcγR, except FcγRI (CD64), and cannot induce CDC or NK cell-mediated ADCC. However, IgG4 mediates ADCP via FcγRI (CD64), FcγRIIa (CD32a) and FcγRIIIa (CD16a) on immune cells. IgG4 undergoes Fab-arm exchange, forming asymmetric, bispecific, and monovalent antibodies. This prevents antigen cross-linking and immune complex formation, allowing IgG4 to block immune responses or target protein activity. Therefore, IgG4 is considered a “blocking antibody” (92). Emerging evidence highlights IgG4’s pro-tumorigenic role in cancer immunity and its immune evasion mechanisms, affecting diagnosis and immunotherapy (89). A unique feature of IgG4 is its ability to interact with other IgGs via its Fc fragment (93), competing with cancer-specific IgG1 for FcR interaction, undermining IgG1-mediated responses.

A cohort study of esophageal cancer observed that higher IgG4-containing B lymphocyte in tumor tissue and serum IgG4 levels were positively associated with more aggressive tumor growth (61). Nivolumab is an IgG4-based antibody with a stabilized S228P mutation, commonly used in cancer immunotherapy. Interestingly, it has been found to promote cancer growth in mice. Specifically, the topical application of IgG4 accelerated the growth of breast and colorectal cancers, as well as carcinogen-induced skin papillomas (61), suggesting a potential link between IgG4 and rapidly progressive disease associated with cancer immunotherapy. Colorectal cancer (CRC) patients exhibit high serum IgG4 levels, which polarize macrophages into an alternatively activated tolerogenic M2b phenotype (57), promoting an immunosuppressive microenvironment. Consequently, high IgG4 levels in CRC impair anticancer effector cell functions and synergize with macrophages to enhance immune suppression. IgG4 promotes colon cancer progression by inducing tolerogenic M2-like macrophages via FcγRI and PI3K/AKT/STAT3 signaling, leading to an immunosuppressive tumor microenvironment (51). In melanoma, elevated serum IgG4 levels and increased IgG4^+^ B cells have been associated with disease progression and poor prognosis, particularly in early-stage patients (83). Recent research has revealed significant increases in both glutathione (GSH) and IgG4 in the tissue microenvironment of lung, esophageal, and colon cancers. GSH was shown to promote tumor growth by disrupting the disulfide bonds of IgG4 heavy chains, thereby enhancing Fc-Fc reactivity and the inhibition of IgG4 on ADCC, ADCP, and CDC (94). In TNBC, interactions between cancer cell lines and B cells drive class switching to IgG4 in an IL-10-dependent manner, thereby suppressing antibody-driven immune responses. The presence of IgG4+ B cells may serve as an indicator of poor prognosis (71).

Collectively, IgG4 is generally characterized by weak effector functions, including ADCC, ADCP, and CDC, and can competitively interfere with the activity of other antibody isotypes by occupying Fcγ receptors and complement binding sites. Consequently, its presence in the TME is commonly associated with immunosuppressive effects and poor prognosis. Serum IgG4 levels, IgG4+ B-cell levels, and increased IgG4/IgE ratios are significant predictors of disease progression and correlate positively with disease progression and associated with an immunosuppressive tumor microenvironment (**Figure 2**).

### 4.2 IgM

IgM is one of the earliest antibody types to respond to pathogens and possesses a unique structure and function. Typically existing as a pentamer, IgM consists of five monomers and a J-chain linker, forming an asymmetric hexagonal structure that enables interaction with a wide array of receptors and ligands. This structural feature allows IgM to perform diverse roles in immune responses (95). Functionally, IgM plays a pivotal role in the initial immune response to tumors by recognizing and binding tumor-associated antigens, activating the complement pathway, inducing apoptosis, and initiating adaptive immune responses (52).

Mostly, IgM exhibits antitumor effects across various tumors. Researchers demonstrated that IgM exerts cytotoxicity in a complement-dependent manner, as shown by the cytotoxic activity of serum and plasma from healthy donors against the human neuroblastoma cell line Kelly and several melanoma cell lines (75). Additionally, using the human hybridoma technique, thousands of tumor-reactive human monoclonal antibodies isolated from epithelial cancer patients and healthy donors were identified as IgM antibodies, with no IgG or IgA detected. These IgM antibodies bind to carbohydrates on modified tumor-specific receptors, induce apoptosis, and play an essential role in the immune surveillance of human epithelial tumors (52). Specifically, naturally occurring IgM MUC1 antibodies in early-stage breast cancer patients may prevent disease dissemination by targeting circulating or isolated disseminated tumor cells, potentially reducing metastasis and mortality (67). Low IgM levels prior to immune checkpoint inhibitor therapy measured in hepatocellular carcinoma are associated with a poorer prognosis, as evidenced by low OS/PFS. This may be attributed to impaired innate immune activation, reduced antigen clearance, and a weakened immunological baseline (64). By measuring serum IgM antibody levels in stage II melanoma patients before and after surgery, and comparing these levels with disease-free and overall survival, a positive correlation was observed between elevated IgM levels and prolonged survival. These findings suggest that IgM antibodies may exert a tumor-suppressive effect. Elevated IgM antibodies targeting oncofetal antigens in melanoma patients may contribute to improved survival by enhancing immune recognition of tumor-associated antigens, activating complement-mediated cytotoxicity, and strengthening immune surveillance (84). Reduced IgM levels were observed in patients with metastatic melanoma, further supporting the importance of IgM in tumor suppression (85). The antitumor role of IgM in tumors becomes evident through these studies. Moreover, innate-like B cells secrete natural antibodies, primarily IgM, that arise independently of antigen priming and exert defined antitumor functions. These natural IgM molecules contribute to early tumor immunosurveillance by targeting altered self-structures on malignant or stressed cells, activating complement, facilitating opsonophagocytic clearance, and in some settings inducing direct cytotoxicity (96). Notably, L2pB1-derived nIgM suppressed tumor growth in B16F10 melanoma and MC38 colon cancer spheroid models via tumor recognition and lipoptosis induction (97).

However, IgM can also promote tumor growth in certain cancers. The specific mechanisms underlying this pro-tumorigenic role are not fully understood, but it is believed that immunoglobulins (including IgM) secreted by tumor cells may contribute to tumor cell proliferation, migration, immune escape, and inflammatory responses (98). For example, the ablation of FcMR, the putative receptor for soluble IgM, inhibited melanoma growth and extended survival in mice, indirectly indicating a pro-tumorigenic role for IgM. Mechanistically, FcMR impairs mononuclear phagocyte function by promoting tolerogenic phenotypes in dendritic cells and tumor-associated macrophages, thereby suppressing CD8^+^T cell activation and infiltration. These findings suggest that FcMR promotes tumor progression by suppressing antigen presentation and inhibiting effective T cell-mediated immune responses (86). In chronic lymphocytic leukemia, secreted IgM (sIgM) drives tumor progression by inducing the accumulation of myeloid-derived suppressor cells (MDSCs), which inhibit T-cell proliferation and diminish antitumor immune responses (48). In hepatocellular carcinoma, IgM facilitates tumor metastasis through epithelial-mesenchymal transition (EMT), mediated by the polyimmunoglobulin receptor (pIgR). This process promotes the expression of EMT-related transcription factors, such as Snail and Twist1, and activates Smad signaling pathways. Elevated expression of these factors is strongly associated with early tumor recurrence and metastasis (63). Similarly, another study suggested that elevated circulating SIgM in patients with colorectal cancer strongly suggests liver metastasis (53). Patients with metastatic breast cancer were found to have significantly lower IgG levels and higher IgM levels compared to those without metastases, indicating a potential association of IgM with tumor metastasis. Tumor-associated CD19^+^CD39^-^B regulatory cells suppress antibody responses by dysregulating class-switch recombination, resulting in sustained IgM expression while inhibiting IgG production. Elevated IgM levels may contribute to immune evasion by impairing effective antibody-mediated immunity, thereby promoting tumor progression (68).

Overall, IgM exerts context-dependent dual roles within the TME through distinct mechanisms. On one hand, IgM contributes to anti-tumor immunity by mediating CDC, enhancing phagocytosis, facilitating immune surveillance, and inducing tumor cell apoptosis via its polyreactive binding to tumor-associated antigens. On the other hand, IgM may promote tumor progression by driving EMT, expanding immunosuppressive populations such as MDSCs, impairing antigen presentation through FcμR signaling, and fostering immune evasion via persistent IgM expression and inhibited class-switch recombination. These multifaceted and context-specific functions underscore the complexity of IgM-mediated regulation in tumor immunity (**Figure 2**).

### 4.3 IgA

IgA exists as secretory IgA (SIgA) in mucosal immunity, where it serves primarily as the first line of defense, protecting the mucosal surface from pathogens and toxins. In contrast, accumulating evidence indicates that IgA within the TME is frequently linked to immunosuppressive and tumor-promoting activities. In contrast, accumulating evidence indicates that IgA within the TME is frequently linked to tumor-promoting activities.

The tumor-promoting effect of IgA in the TME depends on its interactions with other immune cells and molecules. In HCC, inflammation-induced IgA+ cells directly inhibit cytotoxic CD8+ T cells in the liver by expressing programmed death-ligand 1 (PD-L1) and interleukin-10 (IL-10), thereby promoting tumor progression. This mechanism suggests that IgA+ cells promote tumorigenesis and progression by inhibiting anti-tumor immune responses (65). IgA interactions with cancer-associated fibroblasts (CAFs) play an important role in tumor progression. In HCC, IgA complexes induce the transformation of CAFs into a matrix phenotype and increase the expression of PD-L1, thereby inhibiting the cytotoxic function of CD8+ T cells. This interaction further weakens the anti-tumor immune response, promoting tumor growth and spread (99). IgA also activates inflammatory pathways within tumor cells by binding to pIgR in some tumor types, promoting tumor cell growth and survival. In endometrial cancer, the binding of IgA to pIgR activate inflammatory pathways within tumor cells, enhancing immune recognition and survival of tumor cells (72). IgA blocks cytotoxic T cell responses in melanoma (100), induces tolerance in the gut (101), and has an immunosuppressive role in intestinal transplantation by counteracting IgG activity (102). In-depth analysis of circulating and tumor-resident B cells in metastatic cutaneous melanoma reveals abnormalities in B cell profiles and antibody recombination, with IgA1 playing a role in promoting tumor progression (87). Bulk and single-cell RNA sequencing (RNA-seq) and spatial transcriptome data analysis of human bladder cancer tumor cells, along with ligand/receptor crosstalk quantification using stepwise regression Cox analysis, revealed that IgA in the luminal papillary (LumP) and luminal unstable (LumU) subtypes of bladder cancer promotes tumor progression (79). Reanalysis of single-cell RNA (scRNA) and spatial transcriptomics (ST) data from a publicly available dataset to analyze the correlation between plasma cell infiltration and patient prognosis in the TCGA-STAD cohort showed that higher IgA infiltration correlates with shorter patient survival time (62). In an assay of fresh, frozen, and paraffin-embedded specimens from patients with hepatocellular carcinoma and liver metastases from colorectal cancer, IgA was found to promote the progression of colorectal cancer with liver metastases, though the mechanism remains unclear (54). Analysis of 15,115 immune and non-immune cells from primary tumors and liver metastases of 18 colorectal cancer (CRC) patients, using two single-cell RNA sequencing technologies (Smart-seq2 and DNBelab C4), revealed that IgA promotes tumor progression (58). Measurement of fresh tumor specimens from 100 patients (48 women and 52 men; ages 29–89 years) who underwent surgery for colorectal cancer at Dukes A or B stage revealed that elevated circulating SIgA and SIgM levels strongly suggest hepatic metastases in colorectal cancer patients (53).

Conversely, IgA may also be associated with anti-tumor effects in the TME. In ovarian cancer (42), IgA has been detected on tumor cells co-localized with the pIgR, facilitating IgA transcytosis and sensitizing tumor cells to cytotoxic T cell-mediated killing. IgA produced by immortalized B cells reactive to the N-terminal domain of thrombospondin-1 (TSPN) and brain-derived neurotrophic factor (BDNF) recognizes antigens on ovarian cancer cell lines and more effectively inhibits the growth of autologous tumors *in vivo* compared to control IgA (73). Studies have shown that IgA antibodies enter tumor cells via transcellular transport, hinder oncogenic signaling, and promote T cell-mediated cytotoxicity (103). This mechanism is particularly significant in ovarian cancer, where IgA binds to poly-IgA receptors, activates transcriptional changes within tumor cells, and inhibits the RAS pathway, thereby enhancing T cell-mediated killing and hindering tumor progression (73). Tumor-derived B cell IgA redirects myeloid cells, such as neutrophils and macrophages, to target tumor cells by binding to tumor antigens, causing tumor cell death (73). Screening identified BRCA1 and TP53-deficient mouse ID8 OC cell lines for experimentation. TRAF3 in OC cells is an immunosuppressive modulator that down-regulates MHC class I and IFN-I signaling, restricts B-cell activation, and reduces anti-tumor immunity. TRAF3 was found to be involved in the progression of TRAF3KO tumors and significantly higher in the ascitic fluid of these tumors compared to ITB1. B cells were found to be involved in the progression of TRAF3KO tumors, with significantly higher levels of surface-bound and secreted IgA in TRAF3KO tumor ascites compared to ITB1 (104). *In vivo* studies showed that IgA EGFR antibodies significantly enhanced macrophage-mediated tumor cell killing by binding to FcαRI (CD89). This antibody demonstrated stronger anti-tumor activity than traditional IgG antibodies in various tumor models, suggesting that IgA has potential applications in tumor immunotherapy (105). In preclinical models of high-risk neuroblastoma, IgA GD2 antibodies effectively kill tumor cells by activating neutrophil-mediated ADCC without inducing IgG antibody-induced neuralgia. This suggests that IgA antibodies have potent anti-tumor effects in certain tumor types while reducing treatment-related side effects (76). Bulk and single-cell RNA sequencing (RNA-seq) and spatial transcriptome data analysis of human bladder cancer tumor cells, along with ligand/receptor crosstalk quantification using stepwise regression Cox analysis, revealed that IgA inhibits tumor progression in the basal/squamous (Ba/Sq) subtype of bladder cancer (79). Analysis of eight single-cell RNA sequencing (scRNA-seq) datasets and three spatial transcript sequencing (ST-seq) datasets from CRC, along with validation by quantitative reverse transcription-polymerase chain reaction and immunohistochemical staining, demonstrated that IgA inhibits colorectal cancer progression. A simulated fasting diet for colorectal cancer patients and mice was used to analyze intratumoral immune cells, revealing that the diet enhances anti-tumor immunity by reducing IgA-producing cells (59). In clinical samples of primary colorectal cancer and colorectal cancer lung metastasis, the IgA-producing intestinal immune network was significantly dysregulated in the lung metastases. Single-cell RNA sequencing and functional studies revealed that Erbin-positive B-cell subtypes play a key role in lung metastasis. Targeting Erbin (gene) greatly inhibited lung metastasis by reducing PD1 expression on IgA+ B cells, promoting IgA+ B cell aggregation, and enhancing tumor cell killing by CD8+ T cells (60).

In summary, the pro-tumor effects of IgA include: 1) IgA inhibits CD8+ T cell function through the expression of PD-L1 and IL-10 and activates inflammatory pathways; 2) Interaction with CAFs enhances immunosuppressive properties and weakens anti-tumor responses. Conversely, the anti-tumor effects of IgA include: 1) IgA facilitates transcytosis, inhibits the RAS pathway, and enhances T cell-mediated cytotoxicity and neutrophil-mediated ADCC; 2) IgA modulates B cell subpopulations and PD-1 expression, promoting CD8+ T cell killing of tumor cells. The complex and context-dependent roles of IgA in tumors highlight its potential as a novel target for cancer immunotherapy (**Figure 2**).

### 4.4 IgE

IgE has been found to promote the growth of skin tumors (74) in tumor tissues and surrounding lesion skin in mouse models, while in rat models and human pathological tissues, IgE has been shown to inhibit the growth of ovarian cancer (74) and the development of epithelial tumors. IgE binding to FcϵRI recruits basophils to inflamed skin, facilitates their migration. In this environment, IgE/FcϵRI signaling promotes epithelial cell growth and differentiation, strongly driving tumor growth in epithelial cells with oncogenic mutations (74). IgE antibodies recruit macrophages through the TNFα/MCP-1 signaling pathway, enhancing the anti-tumor immune response and thereby inhibiting the growth of ovarian cancer (74) (**Figure 2**).

### 4.5 IgD

While some studies suggest that immunoglobulins are expressed in tumor cells and may influence tumor behavior, research has primarily focused on IgG, IgA, and IgM, with fewer studies on IgD. IgD has been found to have pro-tumorigenic effects in Burkitt lymphoma (47), T-cell acute lymphoblastic leukemia (T-ALL) (50), and IgD multiple myeloma (49). However, in T-cell acute lymphoblastic leukemia (T-ALL) (50), it has also been observed to exhibit anti-tumor effects. Although some studies indicate that IgD may function through IgD FcδR in hematologic tumors, such as T-cell acute lymphoblastic leukemia (T-ALL), these studies are still in preliminary stages, and the role of IgD in other tumor types remains inadequately studied and confirmed (50).

## 5 Cancer derived antibodies in the tumor microenvironment

Strikingly, studies have found that tumor cells themselves can express and secrete Igs (8). Growing evidence suggests that various cancer cell types are capable of producing IgG. In the early stage, Qiu et al. identified that IgG is highly expressed in cancer cells, often referred to as oncogenic IgG (106). This IgG exhibits a unique sialylation modification at Asn162 of CH1 and is widely expressed in cancer stem cells of epithelial carcinomas, where it promotes tumor progression via activation of the integrin-FAK signaling pathway (56).

While the exact function of cancer-derived IgG remains unclear, current findings indicate its role in promoting cancer cell proliferation, invasion, and metastasis, as well as its association with poorer clinical outcomes. Blocking tumor cell-derived IgG has been shown to inhibit tumor growth, underscoring its pro-tumorigenic role (106). In colorectal cancer, recombinant expression of the IgG gene in 80 cases, along with analyses of four colon cancer cell lines and a hormonal immunodeficiency model, revealed that IgG synthesized by colorectal cancer cells contributes to tumor growth and progression. Blocking this IgG has been suggested as a potential therapeutic strategy (107). Additionally, colorectal cancer-derived IgG may facilitate invasion and metastasis through interaction with E-calmodulin (108), offering new targets for immunotherapy. In pancreatic cancer, cancer cell-derived IgG promotes tumor progression via unique glycosylation modifications, correlating with poor differentiation, metastasis, and chemotherapy resistance. Similarly, in gliomas, high expression of cancer-derived IgG is associated with poorer OS and disease-free survival (DFS) (9). Further studies revealed that in gliomas, tumor-derived IgG enhances cell proliferation and migration via the HGF/SF-Met or FAK/Src pathways (8, 98). Moreover, cancer cell-derived IgG aids tumors in evading immune surveillance by inhibiting ADCC and CDC (8, 9, 46, 61). It can also directly promote tumor proliferation and migration by binding to integrin α6β4 and activating the integrin-FAK signaling pathway or by maintaining the biological behavior of cancer stem cells, thereby driving prostate cancer progression. These findings highlight the critical role of cancer cell-derived IgG in tumor biology and its potential as a therapeutic target (109).

## 6 Conclusion

Tumor-infiltrating antibodies exhibit highly complex and heterogeneous functions in the TME, reflecting both their anti-tumor and pro-tumor capabilities. Antibodies often dominate anti-tumor immune responses, engaging in mechanisms like ADCC, CDC, and direct tumoricidal activity. However, under specific TME influences, antibodies can support tumor progression by fostering immune evasion and modifying inflammatory responses. The functional dichotomy of these immunoglobulins underscores the multifaceted role of humoral immunity in cancer. Notably, the functional impact of tumor-infiltrating antibodies is largely dictated by their isotypes. IgG, particularly IgG1 and IgG3, is frequently linked to favorable clinical outcomes by promoting ADCC and ADCP, whereas IgG4 may attenuate immune responses and contribute to immune tolerance. IgM serves as a frontline effector in antitumor immunity via CDC, phagocytosis, and apoptotic pathways. In contrast, IgA is often implicated in tumor-promoting immune suppression, IgE demonstrates potential pro-inflammatory antitumor activity, and the role of IgD remains largely elusive. Additionally, tumor-derived IgG promotes tumor growth, metastasis, and immune evasion.

Understanding the isotype-specific contributions of tumor-infiltrating antibodies will be crucial for refining antibody-based biomarkers and immunotherapeutic strategies. Despite recent advances, the current understanding of antibody-mediated regulation within the TME remains incomplete. The diversity of antibody subtypes, their spatial distribution, and their dynamic interactions with immune and tumor cells present significant challenges. Additionally, the emerging role of tumor-derived antibodies, such as cancer-specific IgG, highlights their potential as novel biomarkers and therapeutic targets. Antibodies secreted by plasma hold immense potential as diagnostic, prognostic, and therapeutic tools in oncology. Their diverse roles in the TME suggest that specific antibody subtypes or patterns could serve as biomarkers for disease progression and treatment response. For instance, the presence of IgG1 and IgG3 isotypes has been correlated with improved outcomes in cancers such as melanoma and non-small cell lung cancer, making them potential prognostic indicators (78). Conversely, IgG4, often associated with immunosuppressive activities, could serve as a marker of poor prognosis or resistance to immune checkpoint inhibitors (89). The identification of tumor-derived antibodies, such as cancer-specific IgG, highlights their role in promoting tumor growth and metastasis, suggesting new therapeutic targets for future interventions. By synthesizing current findings, this review provides a comprehensive understanding of how tumor-infiltrating antibodies regulate tumor immunity, offering insights into their diagnostic, prognostic, and therapeutic potential.

In summary, tumor-infiltrating antibodies represent a frontier in cancer research with vast implications for diagnosis, prognosis, and therapy. Their diverse functions in the TME challenge traditional paradigms and underscore the complexity of humoral immunity in oncology. Bridging current knowledge gaps will require interdisciplinary efforts, combining immunology, molecular biology, and clinical research. Through continued innovation, tumor-infiltrating antibodies have the potential to revolutionize cancer treatment, offering new hope for improved patient outcomes and advancing the field of precision oncology.

## 7 Prospects

The role of a single antibody in tumor progression or suppression can vary significantly across different tumor types, or even within the same tumor type. This duality of function may depend on the specific TME, the presence of regulatory immune cells, and the functional status of immune receptors. In some cases, antibodies may exert tumor-suppressive effects by activating complement-mediated cytotoxicity and enhancing immune recognition of tumor cells. However, in other scenarios, antibodies may contribute to tumor progression by promoting immune evasion, as seen in the dysregulation of antibody class-switch recombination or the persistence of immunosuppressive regulatory B cells. These divergent effects highlight the complexity of the immune response in cancer and suggest that the tumor’s immune landscape, including factors like antigen expression, immune cell composition, and the interaction between the antibody and immune receptors, plays a critical role in determining whether an antibody’s effect is tumor-suppressive or tumor-promoting.

The practical applications of tumor-infiltrating antibodies knowledge are vast. Profiles of these antibodies could serve as non-invasive biomarkers for monitoring disease progression, evaluating therapy effectiveness, or predicting recurrence. Advances in antibody engineering, such as bispecific antibodies and antibody-drug conjugates, provide opportunities for targeted therapies that leverage the unique properties of tumor-infiltrating antibodies. Understanding their spatial distribution and isotype-switching mechanisms could also inform combination therapies. However, significant knowledge gaps remain. The variability of tumor-infiltrating antibodies across different tumors and patient populations complicates universal therapeutic strategies. Additionally, less-studied isotypes like IgD and IgE need further exploration, and tumor-derived antibodies require deeper investigation to better target their mechanisms. Through continued innovation, tumor-infiltrating antibodies have the potential to revolutionize cancer treatment, offering new hope for improved patient outcomes and advancing the field of precision oncology.

## Glossary

TME: Tumor microenvironment
ADCC: Antibody-dependent cell-mediated cytotoxicity
ADCP: Antibody-dependent cellular phagocytosis
CDC: Complement dependent cytoxicity
ICIs: Immune checkpoint inhibitors
Tfh: T follicular helper
Ig: Immunoglobulin
FOB: Follicular B cell
MZB: Marginal zone B cell
Bregs: Regulatory B cells
NK cell: Natural killer cell
BCR: B cell receptor
CSR: Class switch recombination
TGF-β: Transforming growth factor-β
GCs: Germinal centers
AID: Activation-induced cytidine deaminase
S: Switch region
C: constant region
PCs: Plasma cells
SHM: Somatic hypermutation
UPR: Unfolded protein response
TLS: Tertiary lymphoid structures
OS: Overall survival
TNBC: Triple-negative breast cancer
FcγR: Fcγ receptor
NSCLC: Non-small cell lung cancer
TIME: Tumor immune microenvironment
CRC: Colorectal cancer
GSH: Glutathione
SIgM: Secreted IgM
HCC: Hepatocellular carcinoma
MDSCs: Myeloid-derived suppressor cells
EMT: Epithelial-mesenchymal transition
pIgR: Polyimmunoglobulin receptor
SIgA: Secretory IgA
PD-L1: Programmed death-ligand 1
IL-10: Interleukin-10
CAFs: Cancer-associated fibroblasts
RNA-seq: RNA sequencing
scRNA: Single-cell RNA
ST: Spatial transcriptomics
TSPN: Thrombospondin
BDNF: Brain-derived neurotrophic factor
ScRNA-seq: Single-cell RNA sequencing
ST-seq: Spatial transcript sequencing
T-ALL: T-cell acute lymphoblastic leukemia
DFS: Disease-free survival
